# Exploratory factor structure of the neurological evaluation scale in black africans with first episode schizophrenia

**DOI:** 10.1016/j.dib.2015.12.039

**Published:** 2016-01-06

**Authors:** Akin Ojagbemi, Robin Emsley, Oye Gureje

**Affiliations:** a Department of Psychiatry, College of Medicine, University of Ibadan, Nigeria; b Department of Psychiatry, Faculty of Medicine and Heath Sciences, Stellenbosch University, South Africa

## Abstract

While the organization of neurological soft signs (NSS) in schizophrenia into Sensory integration, Motor coordination, and Motor sequencing, is functionally ‘meaningful’, it has not been confirmed by empirical methods such as factor analysis. Data on the exploratory factor analysis of the Neurological Evaluation scale in Black Africans with first episode schizophrenia are presented in this report. Data on the confirmatory factor structure of NSS in this population as well as their interpretation can be found in the work by Ojagbemi et al. (2015) [7].

**Specifications Table**

**Table:** 

Subject area	*Biology*
More specific subject area	*Neurobiology of schizophrenia*
Type of data	*Tables, figure*
How data was acquired	*Clinical (Neurological) examination using the Neurological examination scale(NES)*
Data format	*Analyzed*
Experimental factors	*Medication naïve or minimally treated patients with first episode schizophrenia*
Experimental features	*A wash-out period of one week was allowed for 5 participants (6% of the sample) who had a lifetime exposure to oral antipsychotics of less than 4 weeks.*
Data source location	*Ibadan, South-Western Nigeria*
Data accessibility	*Analyzed data are supplied with this article*

**Value of the data**

• First factor analysis of Neurological Soft Signs (NSS) in Black Africans with schizophrenia.
• Large sample of medication naïve or minimally treated patients with first episode schizophrenia.
• Data provides the possibility for designing a shorter version of the Neurological evaluation scale (NES).
• Data also provides preliminary information about the possibility of unique NSS categories in ethnically and racially distinct populations of patients with schizophrenia.

## 1 Introductory remarks

Neurological soft signs (NSS) are an important subset in the heterogeneous expression of schizophrenia. This is because of their potential as easy-to-measure vulnerability indicators of the disease. The most convincing evidence for this assertion comes from data derived from first episode patients with schizophrenia, or those without prior exposure to antipsychotic treatments [3], [8], [9]. Some evidence have also come from data on first degree relatives of schizophrenia patients [5], [6], as well as patients with schizophrenia spectrum disorders [2], [4].

Factor analysis is a technique used for the reduction of datasets to underlying meaningful sub-sets for the identification of key variables among many observed variables. Globally, existing evidence on the factor structure of NSS in schizophrenia has been mostly based on populations outside the African continent [10], [11], or on samples comprising of Mixed population of Caucasians and other races [3]. Data on the first factor analysis of NSS in antipsychotic naive or minimally treated patients with first episode schizophrenia are presented here.

Data on the confirmatory factor structure of NSS in this population as well as their interpretation can be found in the work by Ojagbemi et al. [7].

## 2 Data

**Fig. 1:**
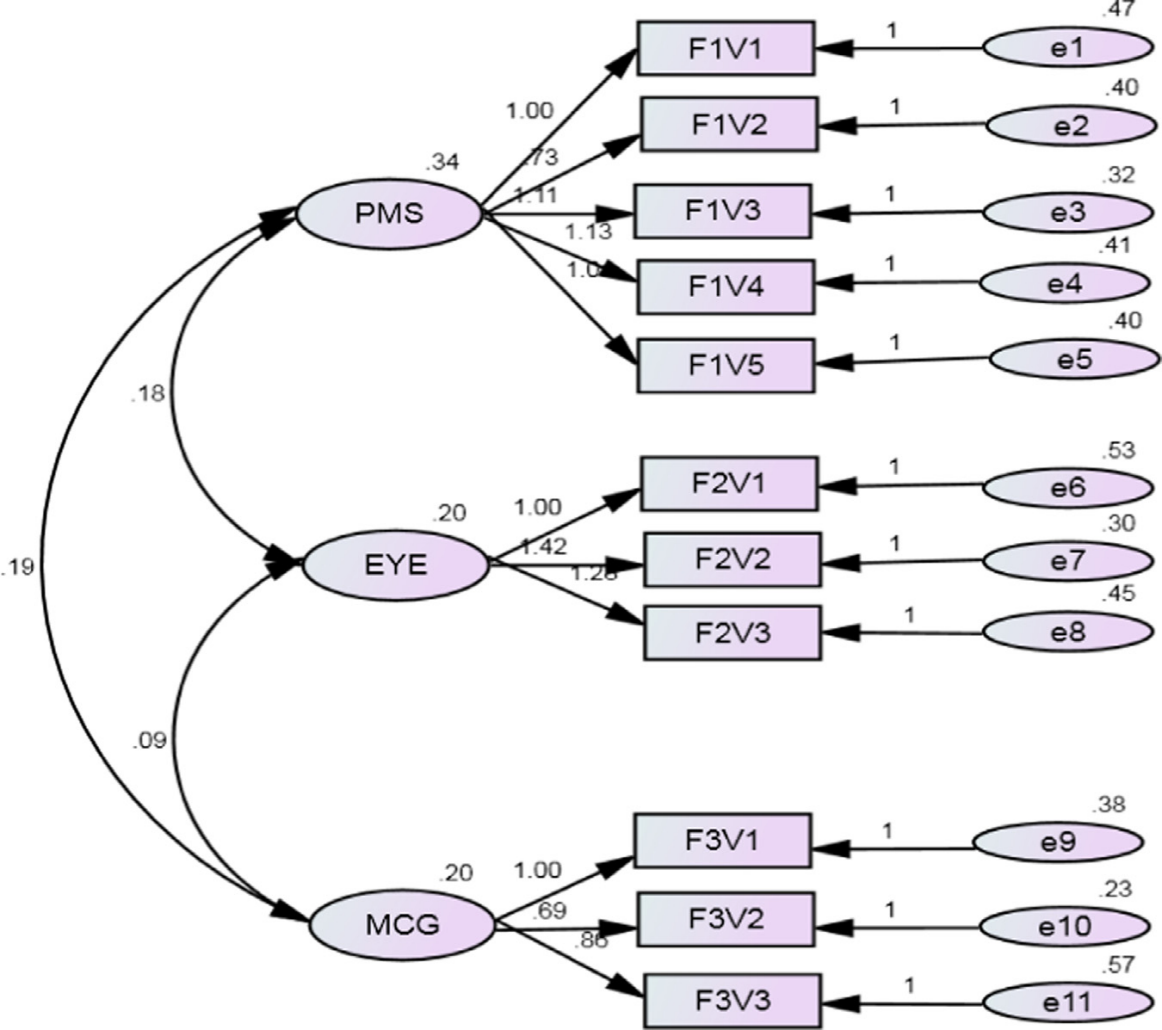
The overall factor structure of neurological soft signs in Nigerians with first episode schizophrenia. **PMS-** Perceptual and motor sequencing, **EYE-** Eye movement, **MCG-** Motor co-ordination and graphaesthesia.

This data is derived from the neurological examination of 84 unmedicated or minimally treated patients with first episode schizophrenia diagnosed according to criteria in the fourth revision of the diagnostic and statistical manual of mental disorders (DSM IV). The data presented in three parts. The first part (see Table 1) contains the proportion of participants with abnormalities of the NSS captured in the Neurological evaluation scale (NES). Also included in Table 1 is the mean and standard deviation (SD) of the severity of individual NSS abnormalities, as well as the overall severity of NSS in the sample. The second part of the data contains the factor loadings, the total variance contributed by each factor loading, as well as their eigenvalues (see Table 2). The mean and SD of the severity of each factor category is also presented. Lastly, the presented data contains the final 3 (correlated) factor structure of NSS in the sample (Fig. 1).

## 3 Experimental design, materials and methods

Patients examined for the purpose of the present data were aged between 16 and 45 years. They presented for biomedical treatment for the first time at the psychiatric units of two general hospitals in Ibadan, Nigeria. They comprised of 47 males and 37 females, consecutively recruited between April, 2009 and June, 2011 (about 26 months). Patients were from the Yoruba ethnic group and were resident within and around Ibadan. Thus, the sample for the present data was racially homogenous.

Patients were approached after they had been seen by the clinician, usually a consultant psychiatrist or a trainee psychiatrist, who was responsible for their routine clinical assessment at presentation. Only those who were assessed by the clinician to have a psychotic illness were approached for possible participation in the study. Written informed consent was obtained from all participants, and/or their guardians after the data collection procedure was explained to them in either English or the local Yoruba language. Neurological soft signs (NSS) in a total of 84 patients with confirmed diagnosis of schizophrenia (81.0%), schizophreniform disorder (17.9%), and schizoaffective disorder (1.2%) according to criteria in the DSM IV were cross-sectionally measured before antipsychotic medications were prescribed.

The NSS were assessed using the NES [1]. The NES includes tests such as tandem walk, rapid alternation movements, finger to thumb opposition, the finger-to-nose test, audiovisual integration, stereognosis, graphesthesia, extinction, and right to left confusion, first-ring test, the first-edge-palm test, the Ozeretski test, and rhythmic tapping test. The other signs assessed by the NES include cerebral dominance, short-term memory, frontal release signs and eye movement. The NES items are scored with reference to the descriptive anchors provided on a three-point scale (no abnormality=0; mild, but definite impairment=1; marked impairment=2) with the exception of ‘suck’ and ‘snout’ reflexes which are scored 0 or 2. A neurological abnormality was defined as the rating of 2 on any 1 item on the NES. The tests were administered by a psychiatrist who had been trained in the use of the NES. Each item was assessed according to a fixed order.

Exploratory factor analysis was conducted on NES items that were abnormal in more than 10% of the entire sample. Items testing for cerebral dominance were excluded from the analyses. Factors obtained following initial maximum likelihood exploration were further rotated using the varimax procedure. Factors are recorded when they have eigenvalues greater than unity, and when they contribute at least 10% to the cumulative variance [11].

## Figures and Tables

**Table 1:** Prevalence and mean Severity scores of NES Items.

**NES Items**	**Number**	**Percent**	**Mean**	**Standard deviation**
**Audiovisual integration**	45	53.6	1.21	0.91
**Fist-edge-palm**	60	71.4	1.56	0.77
**Rythm tapping**	47	56.0	1.29	0.87
**Extinction**	32	38.1	0.91	0.93
**Right/left confusion**	41	48.1	1.19	0.87
**Synkinesis**	22	26.2	0.70	0.86
**Convergence**	22	26.2	0.75	0.85
**Gaze impersistence**	30	35.7	0.93	0.89
**Tandem Walk**	14	16.7	0.50	0.77
**Adventitious flow**	5	6.0	0.24	0.55
**Graphaesthesia**	44	52.4	1.26	0.85
**Stereognosis**	15	17.9	0.51	0.78
**Memory**	63	75.0	1.30	0.82
**Rapid alternating movements**	25	29.8	0.77	0.88
**Finger/thumb opposition**	30	35.7	0.87	0.92
**Mirror movement**	16	19.0	0.51	0.80
**Finger to nose test**	15	17.9	0.57	0.78
**Grasp reflex**	21	25.0	0.50	0.87
**Total NES score**	84	100	11.33	5.85

**Table 2:** Factor structure of the NES Items.

**NES Items**	**Factors**			
**1**	**2**	**3**	**4**
**Audiovisual integration**	***0.6179***	0.3813	0.1862	0.0691
**Fist-edge-palm**	***0.8403***	−0.0635	−0.0507	0.0175
**Rythm tapping**	***0.6279***	0.4463	0.3077	0.0460
**Extinction**	***0.6112***	0.2675	0.2758	0.2573
**Right/left confusion**	***0.5355***	0.1595	0.3305	0.0876
**Synkinesis**	−0.0250	***0.7128***	0.2598	0.2451
**Convergence**	0.1743	***0.7300***	−0.0335	−0.0300
**Gaze impersistence**	0.2665	***0.6417***	−0.1330	−0.1574
**Tandem Walk**	0.1647	−0.0166	***0.6718***	0.0700
**Adventitious flow**	0.1534	0.0558	***0.7188***	−0.0706
**Graphaesthesia**	0.0702	0.3899	***0.5443***	0.2191
**Stereognosis**	0.2321	0.1389	0.0706	***0.8562***
**Memory**	0.4459	0.1318	0.3792	0.3235
**Rapid alternating movements**	0.3139	0.4897	0.1705	0.3702
**Finger/thumb opposition**	0.4290	0.4148	0.2145	0.3418
**Mirror movement**	0.3394	0.3911	0.1305	0.3925
**Finger to nose test**	0.0312	0.1174	0.1409	0.0929
**Grasp reflex**	0.3895	0.2048	0.1155	−0.6424
**Explained variance (%)**	18.1	15.5	10.4	10.1
**Eigenvalues**	6.5	1.5	1.3	1.2
**Severity. Mean (SD)**	6.44 (3.27)	2.38 (2.02)	2.00 (1.56)	0.51 (0.78)

## References

[1] Buchanan R.W., Heinrichs D.W. (1989). The Neurological Evaluation Scale (NES): a structured instrument for the assessment of neurological signs in schizophrenia. Psychiatry Res..

[2] Chan R.C., Wang Y., Zhao Q., Yan C., Xu T., Gong Q.Y., Manschreck T.C. (2010). Neurological soft signs in individuals with schizotypal personality features. Aust. N Z. J. Psychiatry.

[3] Emsley R., Turner H.J., Oosthuizen P.P., Carr J. (2005). Neurological abnormalities in first-episode schizophrenia: temporal stability and clinical and outcome correlates. Schizophr. Res..

[4] Kaczorowski J.A., Barrantes-Vidal N., Kwapil T.R. (2009). Neurological soft signs in psychometrically identified schizotypy. Schizophr. Res..

[5] Mechri A., Bourdel M.C., Slama H., Gourion D., Gaha L., Krebs M.O. (2009). Neurological soft signs in patients with schizophrenia and their unaffected siblings: frequency and correlates in two ethnic and socioeconomic distinct populations. Eur. Arch. Psychiatry Clin. Neurosci..

[6] Neelam K., Garg D., Marshall M. (2011). A systematic review and meta-analysis of neurological soft signs in relatives of people with schizophrenia. BMC Psychiatry.

[7] Ojagbemi A., Akpa O., Esan O., Emsley R., Gureje O. (2015). The confirmatory factor structure of neurological soft signs in Nigerians with first episode schizophrenia. Neurosci. Lett..

[8] Ojagbemi A., Esan O., Emsley R., Gureje O. (2015). Motor sequencing abnormalities are the trait marking neurological soft signs of schizophrenia. Neurosci. Lett..

[9] Prikryl R., Ceskova E., Tronerova S., Kasparek T., Kucerova H.P., Ustohal L., Venclikova S., Vrzalova M. (2012). Dynamics of neurological soft signs and its relationship to clinical course in patients with first-episode schizophrenia. Psychiatry Res..

[10] Sanders Allen, D. N., S, D. F, Tarpey T., Keshavan M.S., Goldstein G. (2005). Confirmatory factor analysis of the Neurological Evaluation Scale in unmedicated schizophrenia. Psychiatry Res..

[11] Sewell R.A., Perry E.B., J.R., Karper L.P., Bell M.D., Lysaker P., Goulet J.L., Brenner L., Erdos J., D׳souza D.C., Seibyl J.P., Krystal J.H. (2010). Clinical significance of neurological soft signs in schizophrenia: factor analysis of the Neurological Evaluation Scale. Schizophr. Res..

