# Supplementary material for: Exploratory factor structure of the neurological evaluation scale in black africans with first episode schizophrenia
Source: Data Brief. 2016 Jan 6;6:471–5. doi: 10.1016/j.dib.2015.12.039 (PMC4716457; doi:10.1016/j.dib.2015.12.039)
Supplement: Supplementary file 1 — Supplementary material [file mmc1.pdf]

COLLEGE OF MEDICINE

UNIVERSITY OF IBADAN
IBADAN, NIGERIA
P.M.B. 5017 (G.P.O.), IBADAN
E-mail: psychiatry@comui.edu.ng

DEPARTMENT OF PSYCHIATRY
UNIVERSITY COLLEGE HOSPITAL
IBADAN, NIGERIA
Telephone: 2410088, 2410109, 2411099, 2413324, 2414946 Ext. 2757
E-mail: psychiatry@comui.edu.ng

Ref. No.

Date:

AUTHORS DECLARATION

On behalf of all authors, I wish to confirm that there are no known conflicts of interest associated with this publication and there has been no significant financial support for this work that could have influenced its outcome.

I confirm that the manuscript has been read and approved by all authors and there are no other persons who satisfy criteria for authorship but are not listed. I further confirm that the order of authorship listed in the manuscript has been approved by all of us.

I confirm that we have given due consideration to the protection of intellectual property associated with this work and there are no impediments to publication, including the timing of publication, with respect to intellectual property. In so doing, I confirm that we have followed the regulations of our institutions regarding intellectual property.

I further confirm that the work covered in this manuscript has been covered by ethical approval by the relevant body and that such approvals are acknowledged within the manuscript.

I understand that the corresponding author is the sole contact for the editorial process. He is responsible for communicating with the other authors about progress, submission of revisions and final approval of proofs. I confirm that I have provided a current, correct e-mail address which is assessable by the corresponding author.

Signed as follows:

Akin Ojagbemi

12/12/2015
